# Prognoses and Long-Term Outcomes of Nasopharyngeal Carcinoma in Han and Uyghur Patients Treated with Intensity-Modulated Radiotherapy in the Xinjiang Autonomous Region of China

**DOI:** 10.1371/journal.pone.0111145

**Published:** 2014-11-06

**Authors:** Ruozheng Wang, Yao Tan, Xiyan Wang, Lingling Ma, Duoming Wang, Yunhui Hu, Yonghui Qin, Kai Liu, Cheng Chang, Jinming Yu

**Affiliations:** 1 Tianjin Medical University, Tianjing, People's Republic of China; 2 Department of Radiation Oncology, The Affiliated Tumor Hospital of Xinjiang Medical University, Urumqi, Xinjiang Uygur Autonomous Region, People's Republic of China; 3 Department of Radiation Oncology, Tumor Hospital of Shandong, Jinnan, Shandong province, People's Republic of China; Institut Pasteur of Shanghai, Chinese Academy of Sciences, China

## Abstract

**Objectives:**

The objective of this study was to investigate the long-term outcomes and prognostic factors for nasopharyngeal carcinoma (NPC) in Han and Uyghur patients treated with intensity-modulated radiotherapy (IMRT) in the Xinjiang region of China.

**Materials and Methods:**

One hundred twenty-one Han and 60 Uyghur patients with newly diagnosed NPC without distant metastasis received IMRT at the Affiliated Tumor Hospital of Xinjiang Medical University between 2005 and 2008. The Kaplan-Meier method was used to estimate survival rates, and the log-rank test was used to evaluate differences in survival.

**Results:**

Comparing Han and Uyghur patients, the 5-year overall survival (OS), disease-free survival (DFS), local control (LC), regional control (RC), and distant metastasis-free survival (DMFS) rates were 81.9% vs 77.6% (*P* = 0.297), 72.1% vs 65.6% (*P* = 0.493), 88.3% vs 86.5% (*P* = 0.759), 95.0% vs 94.6% (*P* = 0.929), and 79.1% vs 75.2% (*P* = 0.613), respectively. Multivariate Cox proportional hazards regression identified the following independent prognostic factors in Han patients: N stage (*P = 0.007*) and age (*P* = 0.028) for OS, and age (*P* = 0.028) for DFS. OS differed significantly between Han and Uyghur patients >60 years old group (*P* = 0.036). Among Uyghur patients, the independent prognostic factors were age for OS *(P* = 0.033), as well as N stage (*P* = 0.037) and age (*P* = 0.021) for DFS. Additionally, Uyghur patients were less likely to experience mucositis and dermatitis than Han patients.

**Conclusion:**

Han and Uyghur patients with NPC had statistically significant differences in age, smoking history, and N staging. There was no significant difference in overall treatment outcomes with IMRT between these 2 ethnic populations in Xinjiang, China.

## Introduction

Nasopharyngeal carcinoma (NPC) is among the most common malignancies in China, especially in southeast coastal areas. Studies have demonstrated that NPC has unique pathogenic factors, such as genetic susceptibility, Epstein-Barr virus (EBV) infection, chemical carcinogens, and environmental factors. Patients with NPC often share common features, such as ethnic susceptibility, family history, and regional aggregation [1].

NPC commonly demonstrates extensive invasion of adjacent tissues with poorly defined and large tumors in close proximity to critical structures, such as the brain stem, spinal cord, and optic chiasm. These features of NPC can complicate adequate surgical resection. Chemotherapy alone has not been very effective. Radiotherapy is generally accepted as the first-line therapy for NPC. Studies have demonstrated that intensity-modulated radiation therapy (IMRT) can substantially concentrate the radiation dose to target volumes while avoiding or reducing unnecessary radiation to normal tissues and organs, thereby leading to gains in the therapeutic ratio. As obtained using IMRT, the differential dose painting to multiple target volumes may also confer a better radiobiological effect. Previous studies have demonstrated that the implementation of IMRT improves quality of life for patients with NPC, in addition to tumor control and survival [2], [3].

Xinjiang Uyghur Autonomous Region is a multinational area with 13 ethnic groups. Uyghur and Han residents constitute the major ethnic populations in Xinjiang [4]. Uyghur and Han populations vary in terms of genetics, lifestyles, and dietary habits. Uyghur and Han residents also tend to use different languages and/or dialects. Accordingly, cases of NPC may differ between Uyghur and Han patients, in terms of both disease and patient characteristics. Therefore, we sought to investigate and compare clinicopathologic factors and outcomes in Uyghur and Han patients who were diagnosed with NPC and received IMRT in Xinjiang, China. These aims were accomplished in the present study.

## Patients and Methods

### Subjects

We collected and analyzed the medical records of 181 patients who had NPC without distant metastasis. All patients were treated with IMRT at Xinjiang Tumor Hospital between January 2005 and November 2008. This study was approved by the institutional ethics committee of The Affiliated Tumor Hospital of Xinjiang Medical University. All patients provided written consent for inclusion in this study.

All patients had pathologically confirmed NPCs. The routine pretreatment evaluations included a complete medical history, physical examination, dental examination, hematologic and biochemical profile, chest radiography, abdominal ultrasonography, and magnetic resonance imaging (MRI) or computed tomography (CT) of the head and neck. All patients had Karnofsky Performance Status scores of at least 80 and adequate renal and liver functions. Data on the patients' general histories were also collected. A patient was identified as Han provided the patient's self-reported family history did not include intermarriage with any other ethnicities in the past three generations and that the patient had lived in Xinjiang or immigrated to Xinjiang. A patient was identified as Uyghur provided that the patient's self-reported family history did not include intermarriage with any other ethnicities in the past three generations, and that the patient had always lived in Xinjiang. Of the 181 patients, 121 were Han (88 men and 33 women, constituting a gender ratio of 2.67∶1), 60 were Uyghur (41 men and 19 women, constituting a radio of 2.16∶1). Patients' ages ranged from 9 to 81 years old, with a mean age of 45 years and a median age of 44 years. None of the patients had World Health Organization (WHO) type I differentiation, 81 had type II differentiation, and 100 had type III differentiation. According to the Union for International Cancer Control–American Joint Committee on Cancer (UICC/AJCC) 2002 staging criteria, 8, 16, 83, and 74 patients had clinical stage I, II, III, and IVa disease, respectively. Comparisons of the clinical characteristics of the Uyghur and Han patients are presented in Table 1.

**Table 1:** Patient characteristics.

Characteristic		Han n (%)	Uyghur n (%)	χ^2^	*P*
Age (year)	Range	11–81	9–67	2.71^a^	0.052
	Median	45	43		
Gender	Male	88 (72.7)	41 (68.3)	0.38	0.602
	Female	33 (27.3)	19 (31.7)		
Smoking history	Yes	54 (44.7)	15 (25.0)	6.55	0.010
	No	67 (55.3)	45 (75.0)		
WHO type	II	59 (48.9)	28 (46.7)	0.07	0.791
	III	62 (51.1)	32 (53.3)		
T stage	T1–2	29 (24.0)	14 (23.3)	0.01	0.925
	T3–4	92 (76.0)	46 (76.7)		
N stage	N0–1	55 (45.5)	14 (23.3)	8.32	0.004
	N2–3	66 (54.5)	46 (76.7)		
Clinical stage	I–II	19 (15.7)	5 (8.3)	1.89	0.388
	III	54 (44.6)	29 (48.3)		
	IV	48 (39.7)	26 (43.3)		
Treatment	Radiotherapy	12 (9.9)	11 (18.3)	2.56	0.110
	Radiotherapy + Chemotherapy	109 (90.1)	49 (81.7)		
Total patients		121	60		

a t-test.

WHO, World Health Organization.

### Radiation Therapy

All patients were treated with IMRT. The head and shoulders were immobilized using a thermal plastic mask. A contrast-enhanced planning CT scan (Philips MX8000, Philips Healthcare, Cleveland, USA) was obtained with a 3-mm slice thickness, with coverage beginning from the skull vertex and ending 1 cm below the clavicles. Following the guidelines of the International Commission on Radiation Units & Measurements (ICRU) Reports 50 and 62, the CT images were retrieved on the IMRT workstation to delineate target volumes and normal tissues. The target volumes were defined as follows. The primary tumor volume (GTVnx) and the volume for nodal disease (GTVnd) were delineated by clinically and/or radiographically evident disease. There were 2 clinical target volumes: CTV1 and CTV2. CTV1 included the GTV plus a 5-mm margin, the bilateral nasopharyngeal meatuses, the clivus, the base of the skull, the pterygopalatine fossae, the medial and lateral pterygoids, the nasal cavity, the area behind 1/3–1/2 of the maxillary sinus, part of the ethmoid sinus, the sphenoid sinus, the pharynx clearance, the oropharynx, the upper lymphatic drainage area, and the hyoid. CTV2 included the lower lymphatic drainage area. The planning target volume (PTV) 1 was defined as the CTV1 plus a 5-mm margin, and the PTV2 was defined as the CTV2 plus a 3-mm margin.

Simultaneous modulated accelerated radiation therapy (SMART) was applied. The initial 28 radiation treatments were administered over 5.5–6 weeks at doses of 2.12–2.36 Gy/f (59–66 Gy in total) to the GTVnx, 2.12 Gy/f (59 Gy in total) to the GTVnd, 1.82–2.00 Gy/f (51–56 Gy in total) to the PTV1, and 1.82–2.00Gy/f (51–56 Gy in total) to PTV2. Five additional radiation doses were delivered after the initial treatment: 2.12–2.36 Gy/f (74–78 Gy in total) to the GTVnx, 2.12 Gy/f (70 Gy in total) to the GTVnd, and 1.82–2.00Gy/f (60–66 Gy in total) to the PTV1. The maximum point doses to the adjacent critical organs were restricted as follows: 54 Gy to the brainstem, 45 Gy to the spinal cord, 54 Gy to the optic nerves/optic chiasm, 9 Gy to the lenses, 50 Gy to the hypophysis, 60 Gy to the temporal lobe and temporomandibular joints, and 70 Gy to the mandible. The 50% volume dose to the parotid glands was limited to less than 30 Gy.

A 9-field IMRT comprehensive treatment planning system was used (Eclipse, Varian Medical Systems, Seattle, WA, USA). Dose-volume histograms (DVH) were used to evaluate the treatment plans. The evaluation criteria for the treatment plans were as follows. The volume of PTV that received >110% of the prescription dose was required to be ≤20%. Less than 3% of the PTV could receive <93% of the prescription dose. Outside the PTV, the dose was required to be <110% of the prescription dose. Each case had to be verified for quality control and quality assurance before treatment. A MatriXX evolution system (IBA Dosimetry, Schwarzenbruck, Germany) was used for validation. Patients could receive treatment if the delivery error was less than 5% when compared with the same plan's dose distribution, and as analyzed using OmniPro - IMRT software (IBA Dosimetry).

All patients were re-examined using a flexible nasopharyngoscope and MRI at 3–4 weeks after completion of radiotherapy. Stereotactic Body Radiation Therapy (SBRT) with an X-knife machine (Brainlab AG, Feldkirchen, Germany) was used to deliver an additional boost (dose: 9–12 Gy) for patients with residual disease in the nasopharynx, skull base, or oropharynx. Patients who had >2-cm residual cervical lymph nodes after 3 to 6 months of follow-up underwent interval neck dissections.

### Chemotherapy

Of the 181 patients, 23 were treated with radiotherapy only, and the remaining 158 patients were treated with concurrent chemoradiotherapy, of whom 60 received induction chemotherapy, and 20 received induction plus adjuvant chemotherapy. Concurrent chemoradiotherapy consisted of weekly cisplatin (40 mg/m^2^) for 4 to 6 weeks. However, 2 patients suspended concurrent chemotherapy due to poor tolerance. Induction chemotherapy mainly consisted of 1–4 cycles of cisplatin at 180–100 mg/m^2^ on day 1 and 5-fluorouracil at 800–1000 mg/m^2^ on days 1–5, or cisplatin at 180–100 mg/m^2^ on day 1 and paclitaxel at 135–175 mg/m^2^ on day 1 and the same regimen for adjuvant chemotherapy.

### Follow-up

The duration of follow-up began at the completion of treatment. Acute adverse effects were evaluated weekly during the treatment period. All patients were followed every 3 months through the first 2 years. Each follow-up included basic serum detection, biochemistry profiles, chest X-ray, abdominal ultrasound, and MRI of the nasopharynx, head, and neck areas. Patients who were considered at high risk for distant metastasis received additional CT scans of the chest and abdomen, as well as bone scans. Toxicities were scored according to the toxicity criteria of the Radiation Therapy Oncology Group (RTOG) at each follow-up visit. Follow-ups were carried out by re-examinations, mailings, and/or telephone calls.

### Statistical Methods

SPSS 17.0 software (IBM SPSS, Inc., Chicago, IL, USA) was used for all data analyses. Statistical significance was set at *P*<0.05. Between-group differences were evaluated using the χ^2^ test. Survival time was interpreted as beginning with the initiation of IMRT treatment. Overall survival (OS), disease-free survival (DFS), local control (LC), regional control (RC), and distant metastasis-free survival (DMFS) were estimated using the Kaplan-Meier method. The log-rank test was employed to determine statistical significance. The Kruskal-Wallis analysis for continuous variables was used to compare the distributions of toxicities in different groups.

## Results

### Patient Characteristics

Patient characteristics are presented in Table 1. We observed significant differences between Han and Uyghur patients in terms of smoking history (χ^2^ = 6.55, *P* = 0.010) and N staging (χ^2^ = 8.32, *P* = 0.004). There were no statistically significant differences in gender, pathological classification, T stage, clinical stage, or chemotherapy use between the 2 groups. We found that 84.3% of the Han patients and 91.6% of Uyghur patients had stage III or IV disease.

### Follow-up

**Figure 1:**
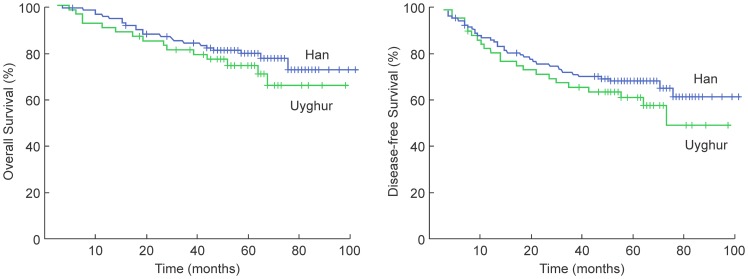
Survival curves for Han and Uyghur patients who received intensity-modulated radiation therapy for nasopharyngeal carcinoma.

As of November 1st, 2012, 10 patients had been lost to follow-up. The median follow-up time was 61 months (range, 1 to 90 months). Overall, 150 patients were followed for more than 3 years, and 92 patients were followed for more than 5 years. The 5-year OS, DFS, LC, RC, and DMFS rates for the entire group were 80.2%, 67.9%, 87.9%, 94.9%, and 77.6%, respectively (Table 2, Figure 1).

**Table 2:** Comparisons of 3-year and 5-year survival rates for Han and Uyghur patients.

		OS (%)	P	DFS (%)	P	LC (%)	P	RC (%)	P	DMFS (%)	P
**3-year**	Han	88.3	0.43	78.2	0.29	88.9	0.66	95.1	0.91	83.5	0.38
	Uyghur	83.2		71.3		92.4		94.8		74.2	
**5-year**	Han	81.9	0.30	72.1	0.49	88.3	0.76	95.0	0.93	79.1	0.61
	Uyghur	77.6		65.6		86.5		94.6		75.2	

OS, overall survival; DFS, disease-free survival; LC, local control; RC, regional control; DMFS, distant metastasis-free survival.

### Outcomes: Death, Recurrence, and Metastasis

Thirty-eight patients died (23 Han and 15 Uyghur patients). Among them, distant metastasis was the cause of death for 5 Han and 8 Uyghur patients. One Han and 3 Uyghur patients died of local recurrence. One Han and 1 Uyghur patient died of second malignancies. Two mortalities among the Uyghur patients resulted from nasal cavity bleeding. The 2 remaining deaths resulted from cardiac disease in a Han patient and an unknown cause in a Uyghur patient.

Overall, 22 patients developed recurrences. The median time of recurrence was 20 months (range, 6–58 months). Among Han patients, there were 9 local recurrence (5 with stage T3 and 4 with stage T4 disease) and 3 regional lymph node recurrences. Two patients developed local and lymph node recurrences. Among Uyghur patients, there were 5 local recurrences (3 with stage T3 and 2 with stage T4 disease) and 3 regional lymph nodes recurrences.

Overall, 34 patients developed distant metastases. The median time to distant metastasis was 13 months (range, 3–72 months). Among Han patients, there were 10 bone, 3 lung, and 5 liver metastases. Four patients developed metastases to multiple organs. Among the Uyghur patients, there were 5 bone, 2 lung, and 2 liver metastases, as well as multi-organ metastases. One patient suffered a parotid lymph node metastasis.

### Analyses of Prognosis

Univariate analyses of the Han and Uyghur patients indicated that age was a prognostic factor for OS in both groups (*P* = 0.031 and *P* = 0.001, respectively) and that N stage was a prognostic factors for OS among Han patients (*P* = 0.043). Patient age (*P* = 0.036) and N stage (*P* = 0.047) were the main factors associated with DFS among Uyghur patients. Among Han patients, T stage (*P* = 0.043) and clinical stage (*P* = 0.020) were the main factors associated with DFS. Furthermore, age and N stage were significant prognostic factors for DFS (Table 3).

**Table 3:** Univariate analysis of OS and DFS for Han and Uyghur patient.

Factors	Han n (%)	Uyghur n (%)	OS		DFS	
			χ^2^	*P*	χ^2^	*P*
Age (years)						
<45	57 (47.1)	34 (56.7)	0.211	0.649	0.009	0.926
45–59	13 (10.7)	19 (31.7)	0.597	0.440	2.031	0.154
≥60	51 (42.2)	7 (11.6)	4.387	0.036	1.177	0.278
N stage						
N_0-1_	55 (45.5)	14 (23.3)	0.560	0.454	1.490	0.222
N_2-3_	66 (54.5)	46 (76.7)	0.321	0.571	1.441	0.230
T stage						
T_0-1_	27 (22.3)	11 (18.3)	1.441	0.230	0.000	0.996
T_2-3_	94 (77.7)	49 (81.7)	1.415	0.234	0.352	0.553
Clinical stage						
I–III	71 (58.7)	32 (53.3)	0.514	0.473	0.138	0.710
Iva	50 (41.3)	28 (46.7)	2.455	0.117	0.254	0.614

OS, overall survival; DFS, disease-free survival.

In log-rank analyses of outcomes with age, N stage, T stage, clinical stage as the strata factors, utcomes differed significantly between the Han and Uyghur groups for patients older than 60 years (*P* = 0.036). However, stratifying by the various levels N stage, T stage, and clinical stage groups did not reveal any statistically significant differences in outcome between the Han and Uyghur groups (Table 3).

### Adverse Events

All patients completed treatment. Two patients had treatment interruptions due to side effects, but eventually were able to complete the treatments after symptomatic management. The primary toxicities were xerostomia, mucositis, dermatitis, and neutropenia. The acute toxicities are summarized in Table 4. There were significant differences between the Uyghur and Han groups in terms of dermatitis and mucositis (χ^2^ = 4.87, *P* = 0.03 for dermatitis; χ^2^ = 9.82, *P* = 0.00 for mucositis). There was no significant difference in terms of xerostomia (χ^2^ = 0.68, *P* = 0.878) or neutropenia (χ^2^ = 0.00, *P* = 0.99) (Table 4).

**Table 4:** Acute toxicities during the treatment of 181 patients with nasopharyngeal carcinoma.

Acute toxicities (RTOG grade) n (%)		0	1	2	3	χ^2^	*P*
Mucositis	Han	-	22 (18.2)	59 (48.8)	40 (33.1)	9.82	0.00
	Uyghur	-	20 (33.3)	32 (53.3)	8 (13.3)		
Dermatitis	Han	-	77 (63.6)	37 (30.6)	7 (5.8)	4.87	0.03
	Uyghur	-	48 (80.0)	10 (16.7)	2 (3.3)		
Xerostomia	Han	10 (8.3)	41 (33.9)	66 (54.5)	4 (3.3)	0.48	0.49
	Uyghur	6 (10.0)	22 (36.7)	31 (51.7)	1 (2.1)		
Neutropenia	Han	45 (37.2)	40 (33.1)	30 (24.8)	6 (5.0)	0.00	0.99
	Uyghur	23 (38.3)	19 (31.7)	14 (23.3)	4 (6.7)		

RTOG, Radiation Therapy Oncology Group.

Kruskal-Wallis analysis.

One hundred and fifty patients survived more than 3 years (105 Han patients and 45 Uyghur patients). The main late toxicity was xerostomia, which had similar incidences in both groups (Table 5). Thirty-eight Han patients (36.2%) and 10 Uyghur patients (22.2%) had grade 1 neck fibrosis. Six Han patients (5.7%) and 2 Uyghur patients (4.4%) had grade 2 or 3 neck fibrosis, but the difference was not statistically significant (χ^2^ = 2.89, *P* = 0.09). Four patients had radiation damage to the temporal lobes, each of whom had skull base tumor involvement prior to the radiation therapy. Three patients had cranial nerve neuropathy. No grade 4 toxicity was observed in this cohort of patients.

**Table 5:** Late toxicities in patients with >3 years follow-up.

Type n (%)		RTOG grade)					
		0	1	2	3	χ^2^	*P*
Xerostomia	Han	35 (33.3)	59 (56.2)	11 (10.5)	-	0.10	0.75
	Uyghur	13 (28.9)	28 (62.2)	4 (8.9)	-		
Hearing	Han	54 (51.4)	46 (43.8)	4 (3.8)	1 (1.0)	0.05	0.83
loss	Uyghur	24 (53.3)	19 (42.2)	2 (4.4)	-		
Neck	Han	61 (58.1)	38 (36.2)	6 (5.7)	-	2.89	0.09
fibrosis	Uyghur	33 (73.3)	10 (22.2)	2 (4.4)	-		
Mandible	Han	100 (95.2)	5 (4.8)	-	-	0.01	0.93
necrosis	Uyghur	43 (95.6)	2 (4.4)	-	-		
Temporal lobe	Han	102 (97.1)	3 (2.9)	-	-	0.05	0.83
necrosis	Uyghur	44 (97.8)	1 (2.2)	-	-		
Cranial nerve	Han	103 (98.1)	1 (1.0)	1 (1.0)	-	0.01	0.91
damage	Uyghur	44 (97.8)	1 (2.2)	-	-		

RTOG, Radiation Therapy Oncology Group.

Kruskal-Wallis analysis.

## Discussion

In China, NPC is among the most common malignancies of the head and neck. According to the WHO International Agency for Research on Cancer, the annual absolute incidence of NPC is 64,796 worldwide, of which 28,022 cases (43.1%) are from China. The regions of China that have high prevalences of NPC are mainly the southern provinces, including Guangdong, Guangxi, Hunan, Jiangxi, Fujian, Yunnan, Guizhou, and Sichuan. It has been reported that the prevalence of NPC is lower in Xinjiang than in Southern China, probably due to differences in lifestyle, environmental factor, climate, and ethnicities [5], [6]. The Affiliated Tumor Hospital of Xinjiang Medical University is the main hospital in this region and has been designated for investigations of the clinical characteristics and prognostic factors of NPC in patients of different ethnicities. Therefore, studies of patients from this hospital may play a critical role in the efforts to curtail NPC in Xinjiang minorities.

The true prevalence of NPC may be obscured by the lack of significant symptoms in early stage disease. It has been reported that 60–70% of newly diagnosed patients have locally advanced disease [7]. As demonstrated in our study, however, 84.3% of Han and 91.6% of Uyghur patients were diagnosed with locally advanced disease. These higher percentages may relate to the differences in the level of health consciousness and economic development in Xinjiang [8]. IMRT has been implemented in our hospital since 2003, which has greatly benefited patients with NPC. IMRT's improved ability to spare normal tissues has allowed us to administer higher doses of radiation for locally advanced disease. When compared with conventional radiotherapy, IMRT has improved quality of life and treatment efficacy.

In a study of 865 patients with NPC, Su et al. [8] observed 5-year OS, DFS, LC, RC, and DMFS rates of 80.2%, 67.9%, 87.9%, 94.9%, and 77.6% respectively. As compared with this report from the southeast coastal region, which has a higher incidence of NPC, we also found similar rates of OS, LC, and RC in the present study. The high proportion of locally advanced disease may contribute to the lower rates of DFS and DMFS in this population. In our study, the 5-year OS, DFS, LC, RC, and DMFS rates were 81.9%, 72.1%, 88.3%, 95.0%, and 79.1% in Han patients and 77.6%, 65.6%, 86.5%, 94.6%, and 75.2% in Uyghur patients, respectively. Although there were no statistically significant differences between the survival rates of these ethnic groups, the percentages provided a non-statistically significant indication that Han patients might have had better survival than Uyghur patients. With longer follow-up periods and a larger sample size of the Uyghur patients, these differences may have been statistically significant. However, additional studies of larger patient cohorts would be necessary to resolve this issue.

In the present study, the age of onset was younger among Uyghur patients than it was among Han patients. We have previously reported a similar result in a study of conventional radiotherapy in 143 patients with NPC [9]. The present study also showed that smoking history differed significantly between Han and Uyghur patients with NPC. As compared with Han patients, a smaller proportion of Uyghur patients were smokers, which may be related to differences in religion, culture, and lifestyle. Previous studies have demonstrated that the frequency of genetic mutations in Han people resembles that in other Asian populations, but significantly differs from that in Caucasians [10]. The frequency distributions of genetic mutations in Uyghur and Kazak people fell between the Asian and Caucasian distributions, but were much closer to the Caucasian distribution. Thus, the characteristics and outcomes of some diseases may differ according to ethnic background.

It has been reported that the pathological type of NPC in high-incidence areas in the inland was mainly WHO III. Particularly, Han et al. found that 98.7% of cases were WHO III type [11]. The percentage of WHO II cases was 71.7% according to a report from Northwest China [12], indicating that there were differences between the pathological distributions of NPC in high- and non-high-incidence areas in China. According to the statistics from Northwest China, the percentages of WHO II and III disease were nearly the same in Han and Uyghur patients (Han, 48.9% and 51.1%; Uyghur, 46.7% and 53.3%). However, the percentage of WHO III NPC was distinctly lower in Northwest China than it was in inland high-incidence areas. The patients in our study were lifelong residents of Xinjiang. As the pathological distribution of NPC patients in Xinjiang differed from the distributions in high-incidence areas, such as Guangdong, Fujian, and Hunan, further studies are warranted to determine whether there are differences in outcomes among the different ethnic populations in China.

We conducted multivariate analyses to identify factors that were independently associated with survival in Han and Uyghur patients with NPC. For Han patients, our analysis revealed that age and N stage were independent prognostic factors for OS, and that T stage and clinical stage were independent predictors of DFS. For Uyghur patients, age and N stage were independent prognostic factors for OS, and age and N stage were independent predictors for DFS. Gender, smoking history, T stage, and clinical stage did not have significant associations with OS or DFS in either Han or Uyghur patients. Further analysis showed that OS was better in Han patients older than 60 years; this difference was statistically significant (*P* = 0.036). However, N stage, T stage, and clinical stage did not differ significantly between the two groups. Previously, Yeh et al. [13] reported that age was independently associated with survival. In a study of 990 cases of NPC in elderly patients, Sze et al. [14] reported that age was an important and independent prognostic risk factor. In the present study, we found that age was independently associated with OS and DFS for both Han and Uyghur patients. This may indicate that elderly patients would have a poor prognosis regardless of ethnicity, perhaps as a result of differences in endocrine metabolism and tissue repair capacity [15]. In Han patients, N stage was associated with OS, but did not have an obvious influence on DFS. In Uyghur patients, age was associated with OS, and age and N stage were associated with DFS. The elderly Uyghur patients showed poor OS, which may be associated with their different attitudes towards ongoing treatment in the face of tumor recurrence or metastasis.

Our conclusions regarding gender and smoking history were not entirely consistent with some previous reports. Indeed, it has been reported that women with NPC had a better prognosis than men with NPC [16], [17], [18]. Further, Xue et al. [19] reported that the risk of developing NPC was 1.6 times higher in smokers than that in non-smokers. In the present study, the OS and DFS were higher for non-smoking and female Han patients. Among the Uyghur patients, women had better OS and DFS than men, as was seen among the Han patients. However, these differences were not statistically significant, perhaps because of the limited sample size.

In a prospective study of 323 cases of NPC, Lin et al. [20] reported that T stage was not a prognostic factor in patients who received IMRT. However, N stage was a significant predictor of DMFS and OS. Our multivariate analysis did not suggest that T stage or clinical stage were independently associated with the survival rate. While the sample size of our study was small, IMRT has overcome some of the deficits of conventional radiation therapy by establishing a more accurate dose distribution with improved conformity to the treatment targets. These benefits of IMRT may have led to better treatment outcomes for our patients with locally advanced disease. IMRT could have mitigated the adverse effects that T stage and clinical stage would otherwise have had on survival. In the present study, however, N stage was a significant independent prognostic factor for OS among Han patients, and was almost significant among Uyghur patients (*P* = 0.064). As previously mentioned, this lack of statistical significance may relate to the relatively small number of Uyghur patients in this study. There were also differences in the adverse events experienced by Han and Uyghur patients. Both oral mucosal and skin reactions were less common in Uyghur patients than they were in Han patients, but the causes of these differences require further study.

Overall, the treatment of NPC at our hospital has greatly improved since the launch of IMRT. Both Han and Uyghur patients obtained excellent outcomes with long-term follow-up. The survival rate has improved significantly, as compared with previous findings [11]. In this study, the distribution of pathological types differed from that in high-incidence areas in China. We also observed some differences between Han and Uyghur patients in terms of acute skin and mucosal reactions and the OS of the elderly Uyghur patients. Further studies should be performed to investigate the possibility of genetic differences in NPC between the patients in Xinjiang and other high-incidence inland regions of China. Additionally, further research may elucidate differences in NPC among other ethnic groups.
